# Risk factors and prognosis of postoperative acute myocardial infarction in elderly hip fracture patients combined with coronary heart disease

**DOI:** 10.1186/s13018-024-04757-1

**Published:** 2024-05-21

**Authors:** Saidi Ran, Qili Yu, MingMing Fu, Zhiyong Hou, Zhiqian Wang

**Affiliations:** 1 Department of Geriatric Orthopedics, The Third Hospital of Hebei Medical University, Shijiazhuang, 050051 Hebei People’s Republic of China; 2 Department of Orthopaedic Surgery, The Third Hospital of Hebei Medical University, Shijiazhuang, 050051 Hebei People’s Republic of China

**Keywords:** Coronary heart disease, Acute myocardial infarction, Hip fracture, Elderly patients, Prognosis, Prediction model

## Abstract

**Introduction:**

This article mainly studies the risk factors for postoperative acute myocardial infarction (AMI) in elderly hip fracture patients combined with coronary heart disease (CHD), constructs a prediction model, and evaluates the prognosis of all the patients.

**Methods:**

This article retrospectively collected elderly patients with hip fracture and CHD who underwent hip fracture surgery at the Third Hospital of Hebei Medical University from January 2019 to December 2021. Demographic data, laboratory indicators, and imaging examination results were collected from the medical case system. The risk factors of postoperative AMI were determined by univariate and multivariate logistic regression, and a nomogram prediction model was established. The ROC curve, calibration curve and DCA decision curve were plotted by R language software. The patients in the training set were followed up for 2 years to evaluate their survival situation.

**Results:**

1094 eligible patients were divided into a training set (n = 824 from January 1, 2019 to September 31, 2021) and a validation set (n = 270 from October 1, 2021 to December 31, 2022). In the training set, women accounted for 58.6%; The average age of the patients was 79.45 years old; The main type of fracture was intertrochanteric fracture. There were 64.7% patients taken B receptor blockers; A total of 166 (20.1%) patients underwent percutaneous coronary intervention (PCI); Hypertension accounted for 55.5%; 520 (63.1%) patients had a preoperative waiting time greater than 3 days; The average hemoglobin value upon admission was 101.36 g/L; The average intraoperative bleeding volume was 212.42 ml; The average surgical time was 2.5 ± 0.3 h; Reginal anesthesia accounted for 29.7%; 63 (68.5%) AMI patients had no obvious clinical symptoms; 68 (73.9%) AMI patients did not show ST-segment elevation in ECG; The risk factors of postoperative AMI were age, hemoglobin at admission, diabetes, chronic kidney disease, intraoperative bleeding, and reginal anesthesia. The AUC of the nomogram prediction model was 0.729. The AUC in the validation set was 0.783. Survival analysis showed a significant statistical difference in 2-year mortality between patients with AMI and without AMI, among all the patients with AMI, patients with ECG ST-segment elevation has higher mortality than patients without ECG ST-segment elevation.

**Conclusion:**

Our research results found that the incidence of postoperative AMI in elderly patients with hip fractures and CHD was 11.1%. Age, diabetes, hemoglobin at admission, regional anesthesia, chronic kidney disease, and intraoperative bleeding are risk factors. The AUC of the nomogram in training set is 0.729. The 2-year mortality rate of the patients with AMI is higher than that of patients without AMI.

## Introduction

Hip fracture is one of the most common fractures in elderly patients. It has a high incidence rate and high mortality, and is often regarded as the last fracture for elderly adults. Pain, bleeding, and stress after the fracture often lead to many complications, such as cardiac complications, acute cerebrovascular disease, and pulmonary infections. Acute myocardial infarction (AMI) is a common acute complication in the perioperative period of hip fractures in elderly adults. Compared with common AMI in internal medicine, the AMI in hip fracture patients often lacks typical clinical symptoms such as chest pain, wheezing, and even changes in ECG. Therefore it increases the difficulty of diagnosis. In addition, compared with symptomatic AMI, asymptomatic AMI has almost as high a mortality rate in the perioperative period of hip fracture as symptomatic patients. Meanwhile, studies have shown that for patients who undergo non-cardiac surgery, the occurrence of perioperative myocardial infarction or myocardial injury increases the all-cause mortality rate by 30 days and 1 year, while the risk of congestive heart failure, nonfatal cardiac arrest, stroke, and 30 day mortality rate is higher [1]. Therefore, it is very important to prevent the occurrence of AMI during the perioperative period.

Perioperative myocardial infarction can be divided into preoperative myocardial infarction and postoperative myocardial infarction. Preoperative myocardial infarction may be related to the underlying diseases of patients such as hypertension and diabetes, which cause coronary atherosclerosis, lumen stenosis, insufficient blood supply to myocardium. Postoperative myocardial infarction is not only influenced by the patient's state at admission, but also by various factors related to surgical process, such as anesthesia type, intraoperative bleeding, intraoperative hypotension, intraoperative hypothermia, vasopressors, intraoperative blood transfusion, and surgical duration. Therefore, compared with preoperative myocardial infarction, there are more factors to consider for postoperative myocardial infarction.

At present, there is limited research on postoperative AMI in elderly hip fracture patients combined with CHD. Previous studies have investigated the prognostic effects of 10 biochemical indicators on predicting postoperative myocardial injury and/or in-hospital mortality in patients with hip fractures [2], but they did not construct the prediction model and take into account of the intraoperative factors. In 2023, Zhang et al. [3] established a prediction model for AMI but it was limited to the range of preoperative. Meanwhile, many studies were limited to the study of AMI in non cardiac surgery. For example, a large international prospective cohort study in 2014 showed a higher risk of AMI after non cardiac surgery [4]. A literature published in the Journal of Clinical Anesthesiology in 2016 mentioned that the apgar score can be used for the evaluation of myocardial injury after non cardiac surgery [5]. There were few studies related to hip fracture surgery. Therefore, our aim is to construct a prediction model for postoperative AMI in elderly hip fracture patients with CHD, to help guide clinical practice and reduce the incidence of postoperative AMI. Meanwhile, follow-up studies will be conducted to analyze the prognosis of the patients.

## Materials and methods

### Patients and groups

This study retrospectively collected data from 1732 elderly patients aged 65–95 who were diagnosed with CHD and had hip fractures between January 2019 and December 2022. Among them, 444 patients with multiple fractures, pathological fractures, old fractures, conservative treatment, chronic heart failure, recent acute cardiovascular disease, incomplete clinical data and 194 patients who were lost during follow-up were excluded. We divided the remaining 1094 patients into a training group (n = 824 from January 2019 to September 2021) and a validation group (n = 270 from October 2021 to December 2022). The database was the electronic medical record system for elderly orthopedics at the Third Hospital of Hebei Medical University. The indicators we collected include the basic demographic characteristics of patients: age, gender, fracture type and treatment history of CHD (percutaneous coronary intervention(PCI), thrombolysis, coronary artery bypass graft(CABG)); Medication history of CHD: (use of antiplatelet or anticoagulant drugs, B receptor blocker, ACEI/ARB, statins, Aldosterone antagonists); Comorbidities (hypertension, diabetes, cerebrovascular disease, chronic obstructive pulmonary disease(COPD), chronic kidney disease, liver disease); Examination indicators at admission, such as hemoglobin, albumin; Imaging examination results: left ventricular ejection fraction(EGFR); Surgical related indicators: preoperative waiting time, surgical time, intraoperative bleeding volume, anesthesia method and intraoperative blood transfusion. The follow-up group were consisted of 824 patients from the training set, with a follow-up period from discharge to September 31, 2023. Three clinical doctors inquired about the patient's survival and death status through phone calls to their relatives. Our research had received ethical approval from the Ethics Review Committee of the Third Hospital of Hebei Medical University. This study was in line with the Helsinki Declaration. All patients provided written informed consent forms, and all information related to patients identity was concealed.

### Disease definition

We define AMI as postoperative blood elevated troponin I > 99% of the upper reference limit (0.04 ng/mL) and simultaneously accompanied by at least one situation: (1) new ischemic ECG changes (ST segment elevation or depression, evolutionary Q-wave, T waves symmetric inversion); (2) ischemic symptoms; (3) the abnormal imaging evidence of new myocardial loss or new regional wall motion [6].

### Statistical analysis

We used SPSS 26.0 and R language software as our statistical analysis software. We used mean and standard deviation (SD) to represent continuous variables, while absolute numbers and percentages were used to represent categorical variables. Student t-test or Mann Whitney U-test was used to compare continuous variables. The chi-square test or Fisher's exact test was used to compare categorical variables. In the training set, patients were divided into AMI group and non AMI group, the two groups were compared to discover significant differences. We included indicators with significant differences (*p*< 0.05) into both univariate and multivariate logistic regression analyses to determine independent risk factors for AMI. VIF was used to evaluate the collinearity relationship between these variables. The significance of the correlation was used by odds ratio (OR) and 95% confidence interval (CI). The result of the Hosmer Lemeshow test,*p*> 0.05, indicated that the nomogram prediction model has good fitness. Based on the results of multivariate logistic regression analysis, we constructed a nomogram prediction model for postoperative AMI. The discriminative ability of the prediction model was based on the AUC of the receiver working characteristic curve. We used calibration curves to evaluate the predicted probability and actual probability of the prediction model. We used decision curve analysis to evaluate the clinical application value of prediction model. We used the Kaplan–Meier methods to compare survival rates, while log-rank tests were used to evaluate the disparities.

## Results

### Characteristics of elderly hip fracture patients with or without AMI

**Fig. 1:**
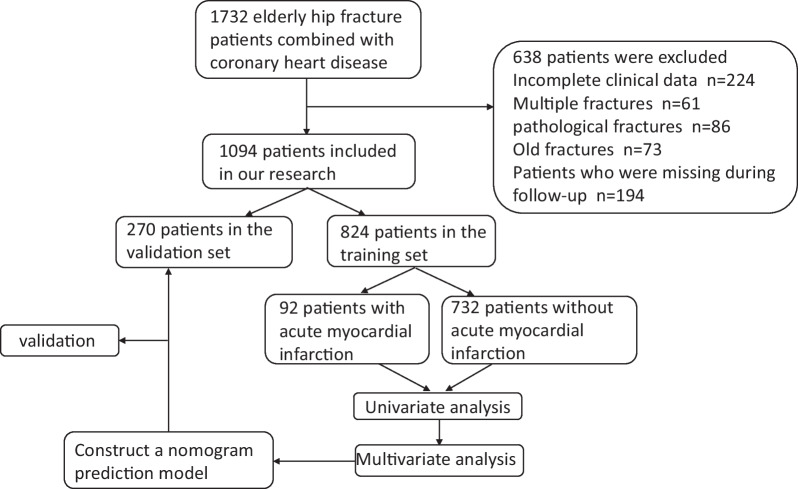
The flow chart of our research

Figure1is the flowchart of our research. In our study, we included 1732 patients age range from 65 to 95 with hip fractures and CHD. After excluding 638 patients who did not meet the inclusion criteria, the remaining 1094 patients were included in our study. We divided them into a training set (n = 824 from January 2019 to September 2021) and a validation set (n = 270 from October 2021 to December 2022). In the training set, 92 patients experienced postoperative AMI. Table1presents the demographic and clinical characteristics of elderly patients with or without AMI in the training set. There were 483 (58.3%) female patients and 341 (41.4%) male patients. There were 446 (54.1%) patients with intertrochanteric fractures and 378 (45.9%) patients with femoral neck fractures. 533 (64.7%) patients took B receptor blockers, 335 (40.7%) patients took aspirin, and 288 (35.0%) patients took statins; A total of 166 (20.1%) patients underwent PCI, 35 (4.2%) patients underwent CABG and 84 (10.2%) patients underwent thrombolysis treatment. Hypertension was the most common complications, accounting for 55.5%. Diabetes and cerebrovascular diseases accounted for 40.2% and 41.1%. 520 (63.1%) patients had a preoperative waiting time more than 3 days. At admission, the average hemoglobin was 101.36 g/L, the average albumin was 32.08 g/L, and the average intraoperative bleeding was 212.42mh. 579(70.3%) patients underwent general anesthesia and 245 (29.7%) patients underwent reginal anesthesia. Table2presents the characteristics of AMI patients. Among 92 postoperative AMI patients, 63 (68.5%) had no significant clinical manifestations, 16 (17.4%) had clinical manifestations of chest pain or tightness, and 13 (14.1%) had dyspnea; 7 (7.6%) patients had hypotension; In terms of ECG, 24 (26.1%) patients were ST-segment elevation, 68 (73.9%) were non ST-segment elevation. In terms of killip rating, 62 (67.4%) patients had a killip rating of 1.

**Table 1:** The demographic and clinical characteristics of elderly patients with or without acute postoperative myocardial infarction

Variable	Total (n = 824)	MI (n = 92)	Non-MI (n = 732)	P(value)
Gender n (%)				
Male	341 (41.4%)	37 (4.5%)	304 (36.9%)	0.810
Female	483 (58.6%)	55 (6.7%)	428 (51.9%)	
Age(years)	79.45	81.27	79.23	0.004*
Type of fracture n (%)				
Femoral neck fracture	378 (45.9%)	38 (4.6%)	340 (41.3%)	0.351
Intertrochanteric fracture	446 (54.1%)	54 (6.5%)	392 (47.6%)	
Medication history n (%)				
Beta blockers	533 (64.7%)	533 (64.7%)	475 (57.6%)	0.727
ACEI/ARB	90 (10.9%)	15 (1.8%)	75 (9.1%)	0.082
Aldosterone antagonists	85 (10.3%)	9 (1.1%)	76 (9.2%)	0.858
Aspirin	335 (40.7%)	36 (4.4%)	299 (36.3%)	0.752
Statin	288 (35.0%)	34 (4.1%)	254 (30.8%)	0.669
CHD treatment history n (%)				
PCI	166 (20.1%)	24 (2.9%)	142 (17.2%)	0.132
CABG	35 (4.2%)	6 (0.7%)	29 (3.5%)	0.251
Thrombolysis	84 (10.2%)	12 (1.5%)	72 (8.7%)	0.340
Comorbidity n (%)				
Hypertension	457 (55.5%)	58 (7.0%)	399 (48.4%)	0.128
Diabetes	331 (40.2%)	50 (6.1%)	281 (34.1%)	0.003*
Old cerebral infarction	339 (41.1%)	47 (5.7%)	292 (35.4%)	0.041*
COPD	169 (20.5%)	21 (2.5%)	148 (18.0%)	0.560
Chronic kidney disease	130 (15.8%)	25 (3.1%)	105 (12.7%)	0.002*
Liver disease	56 (6.8%)	4 (0.5%)	52 (6.3%)	0.327
Preoperative waiting time				
> 3 days n (%)	520 (63.1%)	62 (7.5%)	458 (55.8%)	0.367
Hemoglobin at admission (g/L)	101.36	96.37	101.99	0.000*
Albumin at admission (g/L)	32.08	32.05	32.08	0.949
Left ventricular ejection	61.24	60.90	61.29	0.051
Fraction (%)				
Intraoperative bleeding(mL)	212.42	269.46	205.25	0.000*
Operation time(days)	2.5 ± 0.3	2.4 ± 0.2	2.5 ± 0.4	0.607
Intraoperative blood transfusion n (%)	124 (15.0%)	20 (2.4%)	104 (12.6%)	0.057
Type of anesthetic				
General anesthesia n (%)	579 (70.3%)	49 (5.9%)	530 (64.3%)	0.000*
Reginal anesthetic n (%)	245 (29.7%)	43 (5.2%)	202 (24.5%)	

*ACEI/ARB*angiotensin-converting enzyme inhibitor/angiotensin-receptor blocker,*PCI*percutaneous coronary intervention,*CABG*coronary artery bypass grafting,*COPD*chronic obstructive pulmonary disease

“*”means*p*< 0.05

**Table 2:** Clinical characteristics of postoperative acute myocardial infarction

*Clinical symptoms*	
No obvious clinical symptoms	63 (68.5%)
Chest pain/tightness	16 (17.4%)
Dyspnea	13 (14.1%)
hypotension	7 (7.6%)
*Killip class*	
I	62 (67.4%)
II	25 (27.2%)
III	3 (3.3%)
IV	2 (2.2%)
*ECG changes*	
ST segment elevation	24 (26.1%)
Non-ST segment elevation	68 (73.9%)

### Univariate and multivariate analysis of risk factors for AMI and construct a nomogram prediction model

**Fig. 2:**
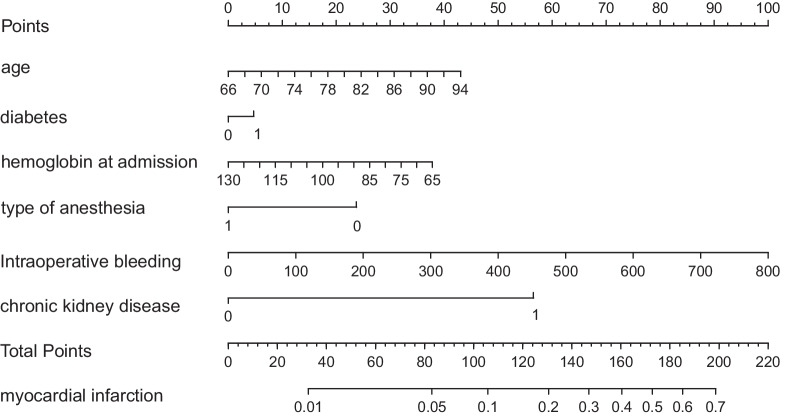
The Nomogram prediction model for postoperative acute myocardial infarction

The univariate and multivariate logistic analyses of the risk factors for AMI in the training set are shown in Table3. From the table, we can find that in the univariate analysis, age, diabetes, old cerebral infarction, chronic kidney disease, hemoglobin at admission, intraoperative blood loss, and reginal anesthesia have significant statistical differences. We incorporated these factors into the multivariate analysis and found that age, diabetes, hemoglobin at admission, reginal anesthesia, chronic kidney disease, and intraoperative bleeding were independent risk factors for postoperative AMI. Multicollinearity suggested that the VIF of all these variables were smaller than 5 so there was no significant correlation between them. We used these risk factors to construct a nomogram prediction model, as shown in the Fig.2.

**Table 3:** Univariate and multivariate logistic regression analysis of risk factors for postoperative acute myocardial infarction in the training set

Univariate analysis					Multivariate analysis		
		OR	95%CL	P	OR	95%CL	*p*
Age (years)		1.054	1.017–1.092	0.004*	1.045	1.006–1.085	0.023*
Chronic kidney disease		2.228	1.346–3.687	0.002*	2.320	1.358–3.963	0.002*
Hemoglobin at admission		0.972	0.957–0.981	0.000*	0.974	0.958–0.989	0.001*
Diabetes Old cerebral infarction Anesthesia Intraoperative bleeding		1.911 1.574 2.302 1.003	1.235–2.956 1.019–2.431 1.482–3.577 1.002–1.004	0.004* 0.041* 0.000* 0.000*	1.724 1.227 2.181 1.003	1.073–2.772 0.762–1.976 1.347–3.463 1.002–1.004	0.024* 0.400 0.001* 0.000*

“*”means P < 0.05

### ROC analysis and verification by a calibration curve and decision curve analysis

**Fig. 3:**
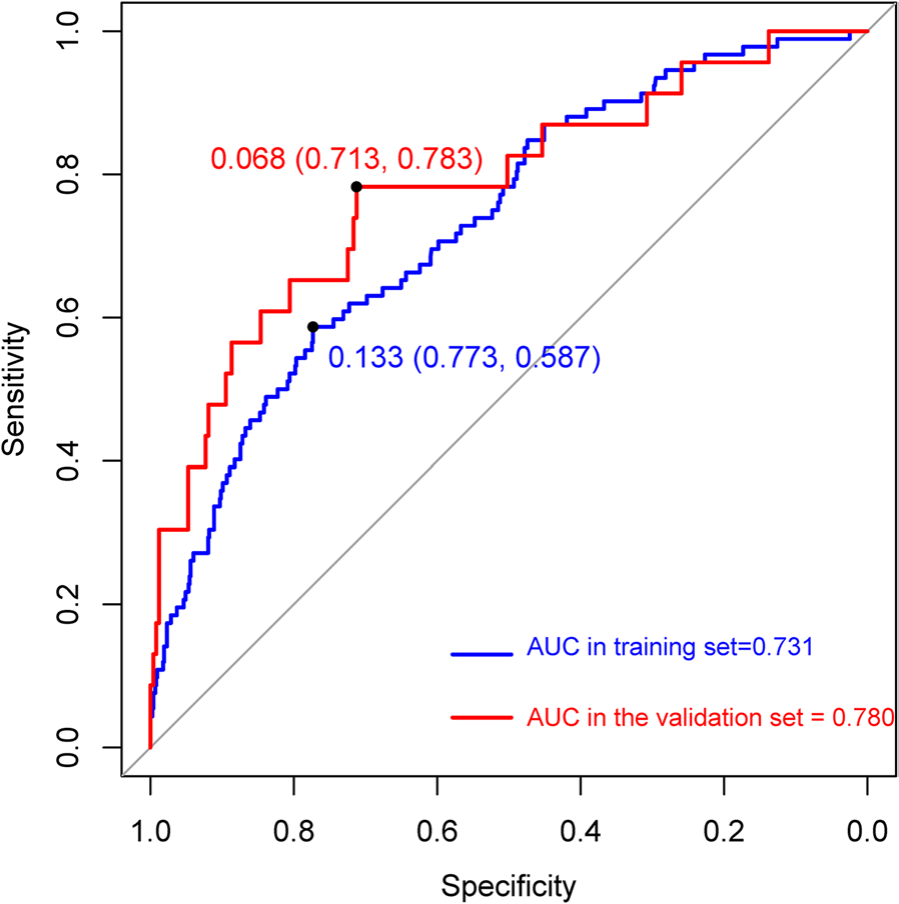
The receiver operating characteristic curves of nomogram in the training set and validation set

**Fig. 4:**
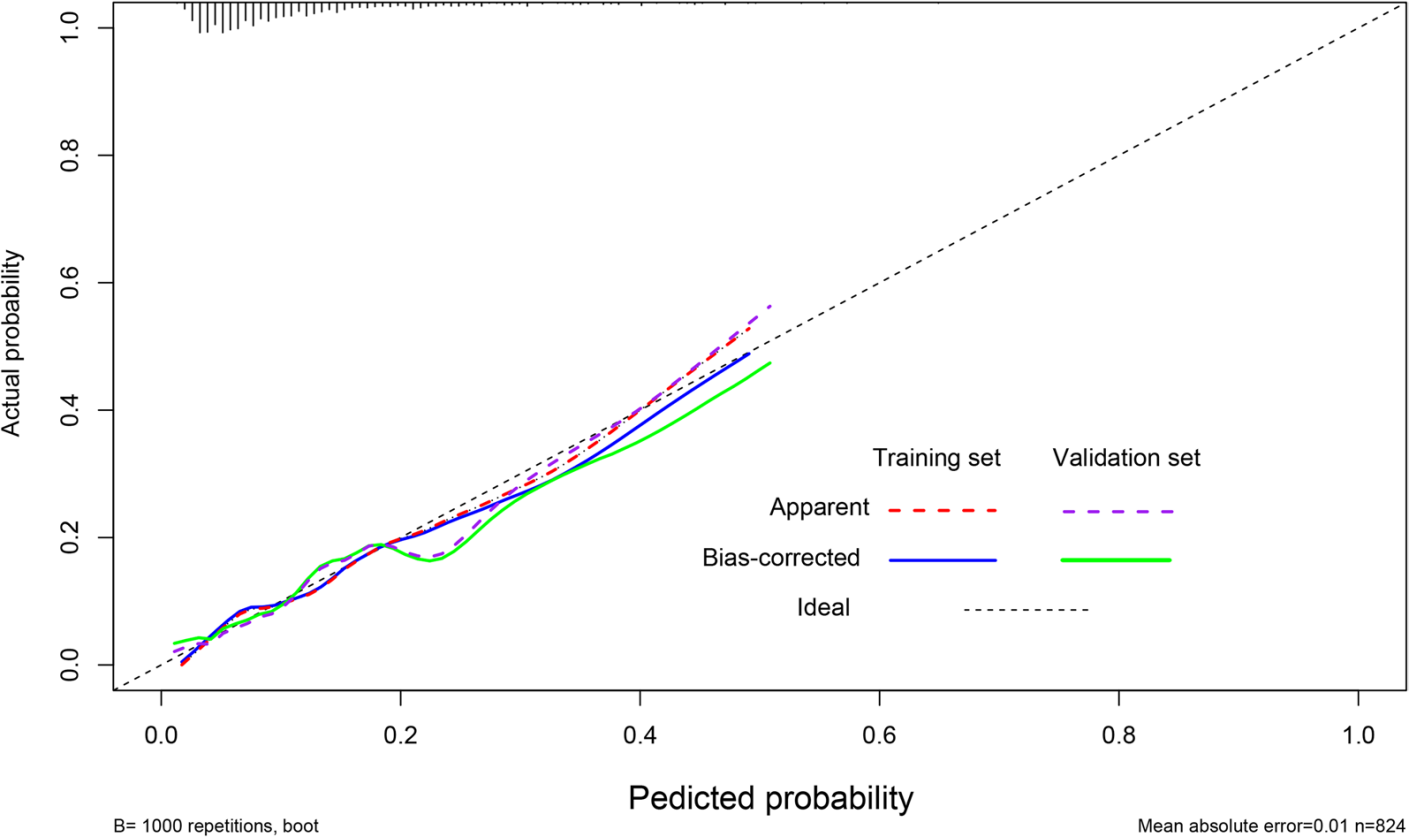
The calibration curve of the nomogram in the training set and validation set

**Fig. 5:**
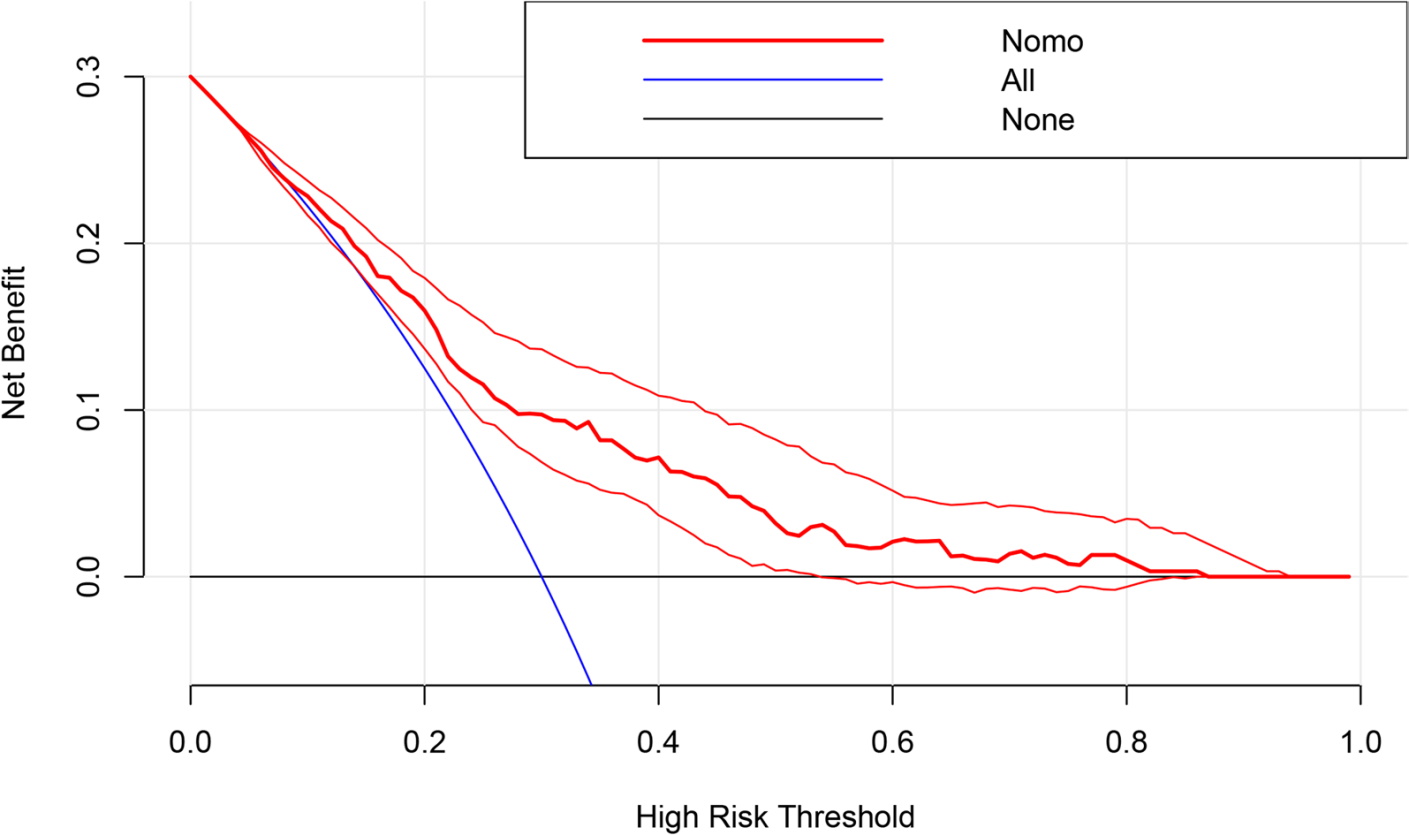
The decision curve analysis of the nomogram in the training set

Figure3shows the ROC curve of the nomogram prediction model in the training set and validation set. With an AUC of 0.729 (95%Cl 0.675-0.783) in the training set and an AUC of 0.783 (95% CI 0.673–0.889). The calibration curves of the training and validation sets are shown in Fig.4, Hosmer–Limeshow goodness of fit test shows good calibration (*p*> 0.05). The decision curve analysis in Fig.5indicates that the nomogram prediction model has good clinical benefits.

### Prognostic research

**Fig. 6:**
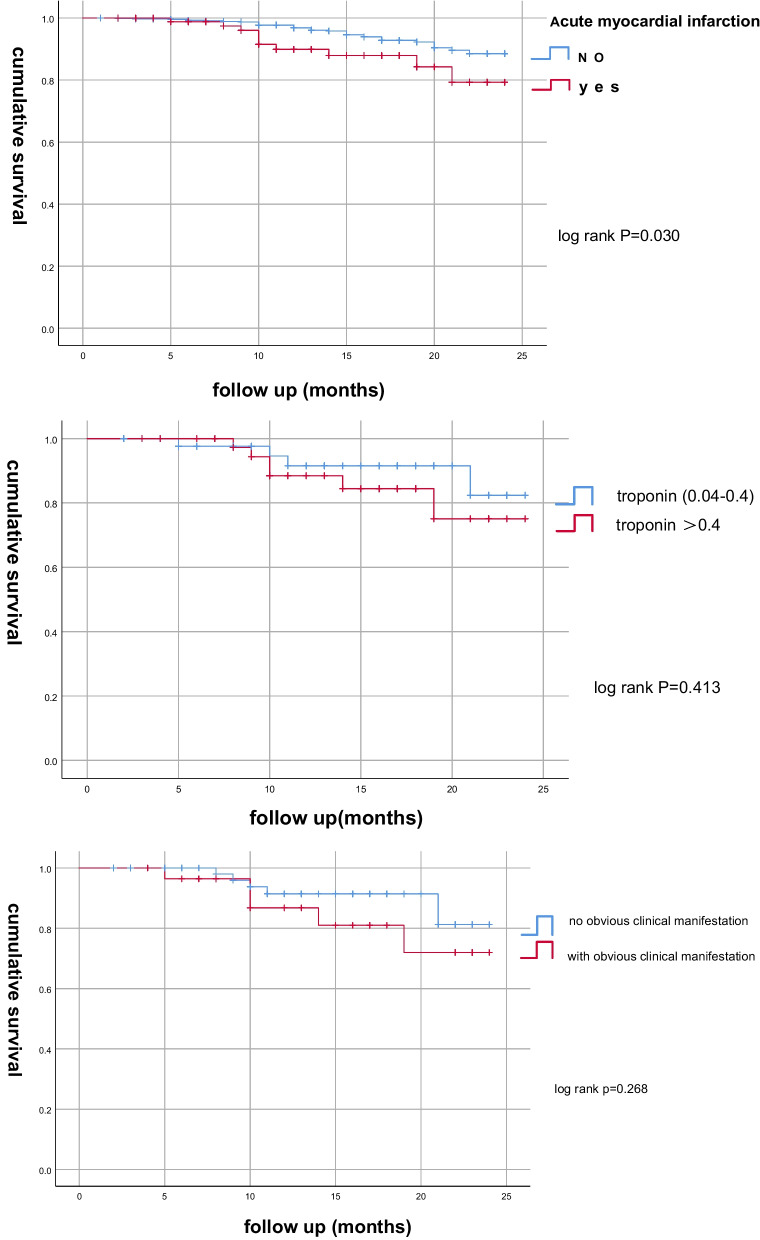

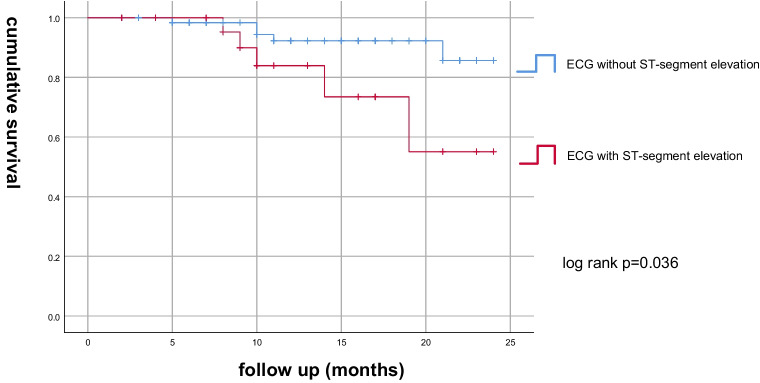
**a**The Kaplan–Meier curve for 24 month mortality rates between postoperative acute myocardial infarction and non myocardial infarction patients. log-rank*p*= 0.030.**b**The Kaplan–Meier curve for 24 months mortality rates in myocardial infarction patients between the group of troponin I > 0.4 and group of troponin I 0.04–0.4. log rank*p*= 0.413.**c**The Kaplan–Meier curve for 24 months mortality rates in myocardial infarction patients between patients with obvious clinical manifestations of acute myocardial infarction and patients without obvious clinical manifestations of acute myocardial infarction. Log-rank*p*= 0.268.**d**The Kaplan–Meier curve for 24 months mortality rates in myocardial infarction patients with ST-segment elevation in ECG and patients without ST-segment elevation changes in ECG. Log-rank*p*= 0.036

Figure6a shows that patients with AMI has lower 2-year survival rate than patients without AMI, the log-rank test is 0.030; Fig.6b shows that among all the patients with AMI, there is no significant statistical differences of 2-year survival rate between patients with troponin over 0.4 and patients with troponin 0.04–0.4, the log-rank test is 0.413. Figure6c shows that among all the patients with AMI, there is no significant differences between patients with obvious clinical symptoms of myocardial infarction and without obvious clinical symptoms, the log-rank test is 0.268. Figure6d shows that among all the patients with AMI, there is significant differences between patients with ECG ST-segment elevation and patients without ECG ST-segment elevation, log-rank = 0.036.

## Discussion

Our research results found that the incidence of postoperative AMI in elderly hip fracture patients combined with CHD is 11.1%, and 68.5% patients have atypical clinical manifestations. Age, diabetes, anemia at admission, reginal anesthesia, chronic kidney disease, and intraoperative bleeding are risk factors for postoperative AMI. Prognostic studies have found that the mortality rate of 2-year in AMI patients is higher than that of non AMI patients. What else, among all patients with AMI, the 2-year survival rate of patients with ECG ST-segment elevation is lower than patients without ECG ST-segment elevation.

Our research found that age is a risk factor for postoperative myocardial infarction in patients with hip fractures. The study by Harris et al. [7] in 2022 suggested that older age was associated with the occurrence of AMI during non cardiac surgery. At the same time, a literature in 2013 also mentioned that the incidence rate of perioperative myocardial infarction increased significantly with age growth [8], which is similar to our research findings. For elderly patients, especially those over the age of 80, they often have weakened immunity, degenerative changes in important organ functions, multiple coexisting diseases and decreased reserve and compensatory abilities. These can lead to reduced tolerance for anesthesia and surgery, resulting in an increase of postoperative complications.

Our study results found that diabetes is a risk factor for AMI after hip fracture surgery. Previous studies have shown that diabetes is generally considered to be a risk factor for AMI during perioperative period of non-cardiac surgery [9]. A literature in 2015 mentioned that diabetes did not seem to be a risk factor for other postoperative complications except for postoperative myocardial infarction [10]. Patients with diabetes often have poor cardiovascular status, coronary stenosis is more serious than patients without diabetes, and they often have multi vessel and multi segment lesions. The operation process including anesthesia, tissue damage, bleeding, hypoxia, will put the patients in a state of stress. These factors can cause the increase of catecholamine hormone, platelet activation and hypercoagulable state, ultimately lead to the occurrence of acute complications such as arterial atherosclerotic thrombosis, coronary spasm, autonomic nervous instability, systemic inflammatory reaction and oxidative stress, finally lead to AMI [10]. For patients with diabetes, as their clinical manifestations are often not obvious when acute complications occur, they should monitor blood glucose in a timely manner, control blood glucose, keep their blood glucose up to the standard, reduce the fluctuation of blood glucose, and perform the coronary artery CT examination when possible. Fully grasp the patient's preoperative condition and prevent postoperative AMI.

Our research results found that preoperative hemoglobin and intraoperative bleeding are also risk factors for postoperative AMI. Different from other research, we treated hemoglobin as a continuous variable. The higher hemoglobin level at admission, the lower probability of postoperative myocardial infarction (OR 0.974 95% CI 0.958–0.989). A research published in JAMA in 2007 suggested that preoperative anemia was associated with an increased risk of postoperative cardiac events in elderly patients undergoing major non cardiac surgery [11]. The 2015 meta-analysis mentioned a correlation between preoperative anemia and postoperative AMI [12]. A retrospective study in 2023 showed that preoperative anemia was associated with the development of postoperative complications, such as cardiovascular events [13]. The mechanism can be explained by a decrease in the amount of hemoglobin in the blood and a decrease in the oxygen-carrying capacity of the blood due to excessive bleeding or malnutrition after a hip fracture, ultimately resulting in myocardial hypoxia; Anemia can also cause tachycardia, increase myocardial oxygen consumption, further exacerbating the imbalance between oxygen demand and supply, finally lead to acute myocardial injury/myocardial infarction. The mechanism of intraoperative bleeding causing AMI is similar to anemia at admission. Jungchan Park et al. in 2021 suggested that intraoperative hemoglobin levels below 7 g/L or less than 50% were defined as significant intraoperative bleeding. Compared to the group without significant intraoperative bleeding, significant intraoperative bleeding group had an increased risk of AMI (OR 1.58, 95% CI 1.43–1.75) [14]. Our results treated intraoperative bleeding as a continuous variable, and the risk of AMI was 1.003 (95% CI 1.001–1.004). The mechanism behind this can be explained as excessive bleeding during hip fracture surgery leading to intraoperative anemia, as well as hypotension and hypoperfusion, ultimately leading to myocardial ischemia, hypoxia, and necrosis. Therefore, for patients who develop anemia at admission, timely blood transfusion and nutritional support treatment should be given. Meanwhile, Jungchan Park et al.'s study mentioned that intraoperative hemoglobin levels associated with myocardial injury after non-cardiac surgery were 9.9 g/dL, and maintaining intraoperative hemoglobin levels above 9.9 g/dL can help prevent postoperative AMI [14].

In our research findings, reginal anesthesia was a risk factor for postoperative AMI. Reginal anesthesia can affect the activity of the sympathetic nervous system, hinder vascular contraction function, and lead to intraoperative hypotension, when blood pressure drops below 65 mmHg, it often affects cardiac perfusion in a short period of time, leading to myocardial injury or myocardial infarction. Research has shown that using an average low dose of 6.5 mg spinal anesthetic can effectively generate intraoperative comfort and motor block, and the incidence of hypotension is lower compared to a high dose of 10.5 mg [15].

Our research findings suggest that CKD was a risk factor for postoperative AMI. A study published in the Journal of Trauma in 2022 showed that CKD patients who underwent hip fracture surgery had a 1.96 times higher risk of cardiovascular events compared to non CKD patients after adjusting for factors such as age, fracture type, and gender (OR 1.96; 95% CI 1.23–3.12), including pulmonary embolism, angina, myocardial infarction, heart failure, arrhythmia, stroke, and death [16]. In our research results, the risk of postoperative AMI in CHD patients with CKD was 2.332 times higher than that of non CKD patients (95% CI 1.383–3.934). CKD and cardiovascular disease have the same risk factors, which includes hypertension and diabetes. Patients with CKD are in long-term of water and sodium retention, the activation of the renin angiotensin aldosterone system (RAAS) and sympathetic nervous system can cause hypertension, leading to left ventricular hypertrophy, left ventricular enlargement, and diastolic dysfunction. In hip fracture surgery, due to factors such as anesthesia, hypotension, inadequate perfusion, anemia, it is more likely to cause myocardial ischemia and hypoxia damage [17]. On the other hand, CKD often leads to vascular calcification and arteriosclerosis [18], resulting in vascular stenosis, insufficient myocardial blood supply, and increased risk of myocardial ischemia, hypoxia, and necrosis after surgery.

Our results found that the incidence of postoperative AMI in elderly patients with hip fractures and CHD was 11.1%. Among these patients, 68.5% had no typical clinical manifestations of chest pain, 26.1% had ECG ST-segment elevation and 73.9% had non ECG ST-segment elevation. Previous studies have mentioned that for patients without CHD, the incidence of perioperative AMI can reach 3%, while for high-risk patients with CHD, the highest incidence can reach 33% [19]. A retrospective case–control study in 2012 showed that 13.8% of elderly patients with hip fractures experienced perioperative AMI, of which 75% were asymptomatic [20]. A 2022 literature mentioned that the incidence of non cardiac perioperative AMI ranges from 0.01 to 10% [7]. These are all similar to our findings. The main mechanism of myocardial infarction in elderly hip fracture patients is type 2 myocardial infarction, caused by an imbalance between myocardial oxygen supply and demand, rather than plaque rupture leading to thrombosis. Our results can also prove this point of view. Advanced age leads to reduced myocardial cell repair ability and increased vulnerability. Both diabetes and CKD can lead to lumen stenosis. Narrow lumen combined with anemia is more likely to cause imbalance between supply and demand, thus causing AMI. Therefore, correcting anemia is the core method of internal medicine treatment for elderly hip fracture patients.

. One study in 2021 showed that patients over 50 years of age with perioperative myocardial infarction had a 1-year mortality rate of 13.9% and a 3-year mortality rate of 21.7% after orthopedic surgery [21]. Our results showed that 1-year mortality was 10.1% and 2-year mortality was 20.7% in patients with AMI, which was significantly higher than 1-year mortality of 3.2% and 2-year mortality of 11.5% in patients without AMI. Many studies have shown that an increase in perioperative troponin concentration is associated with an increase in long-term mortality [22–24]. A 2009 study showed a significant difference in one-year mortality between elderly patients with troponin I > 0.3 and 0.03–0.3 who undergoing emergency orthopedic surgery [25], Chong et al. [26] concluded that troponin I only predicts 1-year mortality, not 2-year mortality, in patients undergoing emergency orthopedic surgery. In our research, there is no significant differences between patients with troponin I levels 0.04–0.4 and troponin I levels > 0.4. We think this may be due to our sample size is small or troponin I level 0.4 is not an appropriate point, which requires further study with large sample size. Meanwhile, our research also found that among AMI patients, patients with ECG ST segment elevation had a higher 2-year mortality rate. It can be explained by the fact that patients without ST segment elevation in ECG have not completely blocked vascular lumens, have smaller area of myocardial necrosis, and can achieve better prognosis after correcting risk factors such as anemia and hypoperfusion. What else, we found that among all patients with AMI, there was no significant difference in 2-year survival between those with significant clinical manifestations and those without, which can be explained by the fact that patients with asymptomatic myocardial infarction are not due to mild condition, what else, these patients often have more comorbidities, such as diabetes, dementia, cerebrovascular diseases, and the use of analgesic and sedative drugs, finally lead to insignificant clinical manifestations. However, such patients also have a higher risk.

### Limitations

Firstly: since this is a retrospective study, some data may have selective bias. Secondly: although the internal validation of the nomogram prediction model demonstrates good discriminability, calibration, and clinical practicality, additional databases are needed for external validation, especially from other distributions. Thirdly: the relatively small number of patients in the AMI group may lead to some controversy in our conclusion. In the future, larger sample size studies are needed to carry out.

## Conclusion

Our research found that the incidence of postoperative AMI in elderly hip fracture patients combined with CHD was 11.1%, and 68.5% of these AMI patients did not have typical clinical manifestations. Age, diabetes, anemia at admission, regional anesthesia, chronic kidney disease, and intraoperative bleeding are risk factors for postoperative AMI. The mortality rate of AMI patients is higher than that of non AMI patients in the follow up of 2 years. Among all AMI patients, there is significant differences between patients with ECG ST-segment elevation and patients without ECG ST-segment elevation.

## Data Availability

The data used to support the findings of this study are available from Zhiqian Wang upon request.
